# Survey dataset on mental health in tech professionals from open sourcing mental health surveys (2017–2021)

**DOI:** 10.1016/j.dib.2024.110377

**Published:** 2024-04-02

**Authors:** Faran Rasheed, Sumra Khan, Syed Muhammad Waqas, Rizwan Ahmed Khan, Sheeraz Arif

**Affiliations:** a Department of Computer Science, Muhammad Ali Jinnah University, Karachi, Pakistan; b Faculty of Information Technology, Salim Habib University, Karachi, Pakistan

**Keywords:** OSMI, Mental health survey, Technology, IT, Mental Illness

## Abstract

The dataset presented here was created by combining surveys conducted by Open Sourcing Mental Illness, a non-profit organization, from 2017 to 2021. The primary objective of the surveys was to assess the prevalence of mental health concerns among individuals employed in the technology sector and to gauge their attitudes toward mental health in the workplace.

The dataset is filtered to include only those respondents with a primary tech role, and descriptive questions are removed, ensuring data consistency and validity of survey responses for effective analysis.

The proposed dataset provides a valuable resource for researchers and practitioners to gain insights into the mental health concerns and attitudes of individuals employed in the technology sector, thus aiding the development of evidence-based interventions and policies to improve the well-being of employees.

Specifications Table

**Table:** 

Subject	Psychiatry and Mental Health
Specific subject area	Survey of mental health illness
Type of data	Table Code in Jupyter Notebook
How the data were acquired	Data was collected from the OSMI (Open Source Mental Illness) organization's surveys on mental illness in the tech industry over the past five years (2017–2021). The data was analyzed and corrected for inconsistencies and filtered based on tech professionals' roles and other factors. The goal of this process was to produce accurate and reliable data that can be used to inform policies and practices related to mental health in the tech industry. It's important to consider any potential limitations or biases in the data set and to be transparent about these limitations in any analysis or reporting of the data.
Data format	Raw Analyzed Filtered
Description of data collection	Data was collected as part of surveys of mental health conducted by OSMI organization.
Data source location	OSMI Survey 2017: https://www.kaggle.com/datasets/osmihelp/osmi-mental-health-in-tech-survey-2017 OSMI Survey 2018: https://www.kaggle.com/datasets/osmihelp/osmi-mental-health-in-tech-survey-2018 OSMI Survey 2019: https://www.kaggle.com/datasets/osmihelp/osmi-mental-health-in-tech-survey-2019 OSMI Survey 2020: https://www.kaggle.com/datasets/osmihelp/osmi-2020-mental-health-in-tech-survey-results OSMI Survey 2021: https://www.kaggle.com/datasets/osmihelp/osmh-2021-mental-health-in-tech-survey-results The data used in this study was obtained from the above links, which are freely available for download upon registration at https://www.kaggle.com. To access the data, interested parties can register for an account on the website and follow the instructions provided. Please note that registration is required to access the data, but there is no cost associated with it.
Data accessibility	Repository name: Survey dataset on mental health in tech professionals from Open Sourcing Mental Health (OSMI) surveys (2017–2021) Data identification number: 10.17632/mmnzx4w8cg.1 Direct URL to data: https://data.mendeley.com/datasets/mmnzx4w8cg/

## 1 Value of the Data

• Useful for identifying trends and patterns in mental illness among tech professionals, which can inform the development of targeted interventions and support systems.
• Beneficial for tech professionals, employers, researchers, policymakers, medical/health organizations, and law enforcement agencies to understand and address mental health needs in the tech industry.
• Can be used to develop experiments that test hypotheses about factors contributing to mental health issues in the tech industry and identify potential interventions or strategies for improving the mental health and well-being of tech professionals.
• Can be leveraged by law enforcement agencies to gain insights into the prevalence of mental health issues among this population and tailor their responses to emergency situations involving individuals with mental health concerns.

## 2 Objective

The OSMI (Open Source Mental Illness) organization is a non-profit that works to support the mental health and well-being of tech professionals. As part of their efforts, they have conducted surveys on mental illness in the tech industry over the past many years. This dataset contains combined, filtered, and corrected responses from the previous five available surveys (2017–2021). The goal of this dataset is to gather data on the prevalence of mental illness among tech professionals, as well as to understand the specific challenges and stressors that tech professionals face and how these may be contributing to mental health issues.

## 3 Data Description

The dataset provided with this submission contains consolidated results of surveys conducted by OSMI from 2017 until 2021, all survey samples conducted during these years, and a supplementary Python Jupyter notebook with all codes used for this explorations.

### 3.1 Dataset Documentation

The dataset is provided as long-form tables in an Excel file. Variable information is provided in Table 1.

**Table 1:** Description of variables contained in the dataset provided.

Variable Name	Description
tech_company	Whether or not the respondent's employer is primarily a tech company/organization
benefits	Whether or not the employer provides mental health benefits as part of healthcare coverage
workplace_resources	Whether or not the employer offers resources to learn more about mental health disorders and options for seeking help
mh_employer_discussion	Whether or not the respondent has ever discussed their mental health with the employer
mh_coworker_discussion	Whether or not the respondent has ever discussed their mental health with coworkers
medical_coverage	Whether or not respondents have medical coverage (private insurance or state-provided) that includes treatment of mental health disorders
mental_health	Whether or not respondents currently have a mental health disorder
mh_share	The willingness of respondent to share mental health illness issues with friends and family
age	Age of respondent
gender	Gender of respondent
country	Country of respondent

Table 2 shows the number of empty rows/records in our combined data. A lot of missing data are due to the changing survey questions across the different years.

**Table 2:** Number of empty rows in combined data initially.

Variable Name	Number of empty responses
tech_company	259
benefits	259
self_employed	0
tech_related_role	259
workplace_resources	259
mh_employer_discussion	259
mh_coworker_discussion	264
medical_coverage	1577
mental_health	0
mh_share	0
age	2
gender	25
country	2

Table 3 shows our dataset with responses for the benefits.

**Table 3:** Responses to ‘benefits’.

Variable Name	Number of responses
Yes	784
I don't know	389
No	290

Table 4 shows our dataset with responses for the age with corrected values.

**Table 4:** Respondents ‘age’ before and after correction.

Age variable	Old value	Corrected value
count	1242	1242
mean	35.00	35.03
std	8.24	8.18
min	0	19
25%	29	29
50%	34	34
75%	40	40
max	66	66

## 4 Experimental Design, Materials and Methods

The aim was to make responses from the previous five years' surveys (from 2017 to 2021) available publicly on the open sourced mental illness (OSMI) website. The survey questions varied slightly over the years, with some new questions added and some changes to the format of the questions.

In order to make the data more relevant and useful, descriptive questions that did not contain meaningful information were filtered out. A handful of questions were slightly different from one another with just the addition of custom HTML tags, like (bold〉, 〈strong〉, etc. Those questions were mapped together. By following this approach, “*Are you self-employed?*” and “<strong>Are you self-employed?</strong>” were considered to be the same question.

Our first step was to either remove empty records or replace them with appropriate values based on the corresponding question. In order to correct the dataset, we planned to address each column individually, following a sequential process.

• **Data Correction - tech_related_role**: Since the objective is to focus on the impact of mental health illness on tech employees, filling empty records with default values will make this analysis biased, hence we only kept those records that have tech_related-role as “true” and discarded other rows with “no” or empty values. This ensured that the resulting data is more focused on the intended population.
• **Data Correction - gender:** We had a small number of records (25) with missing values. To handle these, we assigned the category “Others” to all the empty rows and then divided the resulting data into three groups: Male, Female, and Others. There were a large number of unique responses ranging from “Male” to “mostly male”, “I identify as Man”, and other variations. To standardize these responses, we manually grouped them into three categories. The final dataset included responses from 969 males, 432 females, and 62 individuals grouped into the “Others” category.
• **Data Correction - benefits:** While we formatted and discarded all those records that were not relevant to this dataset i.e. not working in a tech-related company or not having a tech-related job, our empty records were updated and fortunately, we did not have any empty records for the benefits column. But we had different values that users responded to “No”, and “Not eligible” for coverage are mapped to “No” as they both surve the same answer to the question; Does your employer provide mental health benefits as part of healthcare coverage? Table 3 shows our dataset with responses for the benefits.
• **Data Correction - self_employed:** The data for the “self-employed” column did not have any ambiguities, but all the responses were the same (“No”). This indicated that none of the respondents were self-employed, so we did not need to keep this column in our dataset.
• **Data Correction - age:** Age is a significant variable to consider as it can influence people's experiences, attitudes, and behaviors, and can be used to predict certain outcomes and identify trends or patterns within the data. In this case, we found that some respondents had provided incorrect ages, such as “0.” To correct these errors, we replaced all ages less than 18 and greater than 75 with the mean age of respondents between 18 and 75. The results of this correction are shown in Table 4.
• **Data Correction - medical_coverage:** Whether or not respondents had medical coverage is a very important answer for our dataset, and unfortunately, almost all records were empty. In order to correctly fill the empty records we followed multiple approaches as discussed below.
  ○ ***OCED-library:*** According to OCED records, Germany, Canada, France, Spain, and the Netherlands have 100% records for individuals with mental health coverage with their health benefits [1]. So we updated medical coverage for all the respondents from these countries to “Yes”.
  ○ ***Country Law - UK:*** According to OCED records, the United Kingdom has 100% records of its residents registered to a Medical health provider either public or private, including medical health coverage [1].
  ○ Also, according to the UK healthcare system report, in the UK, healthcare is universal and is paid for by 18% tax of a citizen's income [2]. Hence we can update all values to “Yes” if the respondents live in the UK.
  ○ ***Country Law - USA:*** Once again taking benefit from OCED library, The United States of America, had 90% of individuals registered to health care, which includes Mental health coverage. Hence we can update our records to have around 90% of USA respondents with a “Yes” value for medical coverage.

We had 950 records for USA residents in total and around 33% (315) reported not having medical coverage, our target was to reduce it to 10% as according to OCED, the USA has 90% of individuals with mental illness coverage as part of their medical coverage.

Hence, we generated a random sample from a list of respondents who belong to the US and had “No” in their coverage and updated those to “Yes” to overall make it reflect correct OCED stats.

After all the above transformations, we were still left with 218 missing values from out of 1463 total records, which is around 14%. Since assigning random values to these will make our analysis biased, we dropped these records.

• Data Correction - Rest of the data fields: No ambiguity was seen in the responses from “workplace_resources”, “mh_employer_discussion”, “tech_company”, and “mh_coworker_discussion”. All of these had boolean values in responses i.e. 1.0 or 0.0, so we transformed these values from 1.0 to True and 0.0 to False.

After applying all the above data cleaning techniques, we were able to remove/adjust all missing data, and our overall records were reduced to 1242 responses from the previous five years’ survey (2017–2021).

## Ethics Statements

The authors declare that this work did not involve the use of human subjects, social media data, or experimentation with animals.

## CRediT authorship contribution statement

**Faran Rasheed:** Conceptualization, Methodology, Visualization, Writing – review & editing. **Sumra Khan:** Conceptualization, Methodology, Supervision, Writing – review & editing. **Syed Muhammad Waqas:** Writing – review & editing. **Rizwan Ahmed Khan:** Conceptualization, Methodology, Visualization, Writing – review & editing. **Sheeraz Arif:** Visualization, Writing – review & editing.

## Data Availability

Survey dataset on mental health in tech professionals from Open Sourcing Mental Health (OSMI) surveys (2017–2021) (Original data) (Mendeley Data)

## References

[1] OECD (2017).

[2] Chang J., Peysakhovich F., Wang W., Zhu J. (2011). The UK health care system. UK.

